# Human Factor Risk Modeling for Shipyard Operation by Mapping Fuzzy Fault Tree into Bayesian Network

**DOI:** 10.3390/ijerph19010297

**Published:** 2021-12-28

**Authors:** Yang Liu, Xiaoxue Ma, Weiliang Qiao, Huiwen Luo, Peilong He

**Affiliations:** 1 School of Maritime Economics and Management, Dalian Maritime University, Dalian 116026, China; liuyang0120@dlmu.edu.cn; 2 Public Administration and Humanities College, Dalian Maritime University, Dalian 116026, China; maxx1020@dlmu.edu.cn (X.M.); hepeilong@dlmu.edu.cn (P.H.); 3 Marine Engineering College, Dalian Maritime University, Dalian 116026, China; 4 College of Arts and Sciences, New York University Shanghai, Shanghai 200120, China; yq_wl@dlmu.edu.cn

**Keywords:** human factors, shipyard operation, Bayesian network, fuzzy fault tree, risk assessment

## Abstract

The operational activities conducted in a shipyard are exposed to high risk associated with human factors. To investigate human factors involved in shipyard operational accidents, a double-nested model was proposed in the present study. The modified human factor analysis classification system (HFACS) was applied to identify the human factors involved in the accidents, the results of which were then converted into diverse components of a fault tree and, as a result, a single-level nested model was established. For the development of a double-nested model, the structured fault tree was mapped into a Bayesian network (BN), which can be simulated with the obtained prior probabilities of parent nodes and the conditional probability table by fuzzy theory and expert elicitation. Finally, the developed BN model is simulated for various scenarios to analyze the identified human factors by means of structural analysis, path dependencies and sensitivity analysis. The general interpretation of these analysis verify the effectiveness of the proposed methodology to evaluate the human factor risks involved in operational accidents in a shipyard.

## 1. Introduction

**Figure 1:**
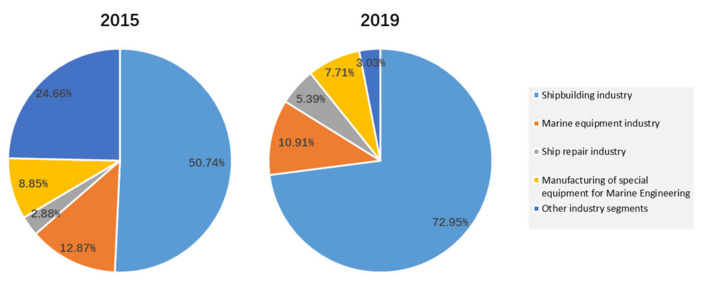
Comparison of shipbuilding industry structure based on the share of business income.

Increasing concern about various risks involved in shipyards, especially the risks that are human-related, can be observed [1,2]. Many dangerous operations are carried out at the same time and in the same space, and the risks on site in a shipyard are highly interconnected and harmful [3]. Therefore, industrial safety management has constantly been explored, but it is difficult to effectively reduce occupational incidents and accidents [4] in a shipyard. In 2018, the revised code of practice for safety and health in shipbuilding and ship repair was adopted at the 329th session of the International Labor Organization (ILO) [5], aiming to help governments, employers and workers address new safety and health problems and provide a reference for the promotion of work safety. Accident investigations have received great attention in Singapore. To prevent or minimize the recurrence of accidents and hazardous events in workplaces such as shipyards, the Attorney-General’s Chambers (AGC) [6] passed a legislative amendment in April 2017, adding provisions on publishing representative accident casualty reports. In addition, under the structural adjustment of the shipbuilding industry around the world, the proportion of the shipbuilding and ship repair industry is increasing compared to that of other sub-industries. According to the statistical data of the China Association of the National Shipbuilding Industry (CANSI), as shown in Figure 1, in 2019 the shares of the shipbuilding industry and ship repair industry accounted for 72.95% and 5.39% of overall business income, respectively. Compared with the statistical results of 2015, the proportion of these two branches increased significantly. With the opportunity of implementation of the 0.5% sulphur limit and the mandatory requirement for installing a ballast water management system (BWMS) on board, the shipbuilding and repair business is expected to benefit. However, the operational risks pertaining to the installation and refit of equipment would require much attention. Therefore, faced with both threats and opportunities, it is important and urgent to reduce the occurrence of occupational accidents in shipyards.

### 1.1. Related Works

A lot of studies have been undertaken to identify and evaluate various causes contributing to the occurrence of accidents in shipyards. Jacinto and Silva [7] studied the causal pathways and their consequences of the accidents that occurred at Arsenal do Alfeite (a large shipyard in Portugal) by bow-tie diagram technique. The 115 fatalities between 2000 and 2010 that occurred in Turkish shipyards were investigated statistically by Barlas [3], and five main causes were identified. A statistical technique is also applied by Ozkok [8] and Romuald Iwańkowicz and Rosochacki [9] who studied the characteristic of the shipyard accidents reported in the years 2000–2005, and the hazards involved in these accidents were identified from technical and ergonomics aspects. Meanwhile, the ship repair yard accidents that occurred between 1989 and 2008 in Greece were collected and analyzed statistically by Fragiadakis et al. [10] with the application of an adaptive neuro-fuzzy inference system (ANFIS) model based on five parameters associated with these accidents. Later, these five important parameters were also utilized by Tsoukalas and Fragiadakis [1] to investigate 284 occupational accidents reported by the Greek Labour Inspectorate Agency by means of integrating multivariable linear regression and genetic algorithms, and the key risk factors involved in these shipyard accidents were mainly related to humans, such as carelessness of workers, erroneous series of human operations, and insufficient safety training, and they also studied the influence of working conditions on occupational injuries. After investigating the reported fatal injuries in shipyards between 2004 and 2014 in Turkey, Barlas and Izci [11] summarized five major causes for these accidents which are listed as falling higher elevation to lower level, exposed to electric shock, fire and/or explosion, being struck by or struck against objects and caught in between, and drowning. In addition, Crispim et al. [12] summarized the potential risks involved in military shipbuilding in Brazil which were then analyzed semi-quantitatively by a visual Delphi and Bayesian network (BN). 

Identification of critical risk events involved in the shipyard operation is an effective way to prevent accidents in shipyards. When identifying the hazardous events or critical risk events involved in shipyard operations, the factors to take into consideration have been proposed by International Labor Organization (ILO) [5]. The questionnaire survey and expert interviews were utilized by Lee et al. [13] to extract the potential critical risk events involved in the shipbuilding industry in Korea, and the potential hazardous events contributing to the occurrence of shipyard accidents were also identified by Zheng et al. [14] with the application of an energy source-based job safety analysis (JSA) technique. Njumo [15] mapped the fault tree analysis (FTA) into formal safety assessment (FSA) to investigate the accident scenario in shipyards. Ozkok [8] applied failure mode and effect analysis (FMEA) to identify the most risky activities and work stations within a shipyard, and later, the FMEA model was also utilized by Efe [2] to determine the most three risky scenarios, which were listed as “lack of nets on ship scaffolds”, “utilization the inappropriate ladders” and “empty fire extinguishers”. In addition, the historical data associated with shipyard accidents are widely used by scholars to identify risk events. Shinoda et al. [16] developed a database containing shipyard occupational accidents and an information processing model to identify the hazardous events; later, Shinoda et al. [17] designed a work observation system based on this database, and similarly, the database containing various resources aimed at risk control in shipyard was also utilized by Cebi et al. [18] to establish a web-based decision support system to aid decision-making for risk management. Based on the obtained historical data, the BN is often applied to identify the critical risk events in the shipbuilding and repair industry; for instance, Lee et al. [19] developed a BN to implement risk evaluation with the help of questionnaire approach, and then, Basuki et al. [20] used the probabilistic-based approaches to quantify the parameters involved in BN model to analyze the critical risks in the shipyard industry. The BN model was also applied by Costa et al. [21] to investigate the underlying causal factors involved in shipyard accidents. To identify the critical risk factors (CRF) involved in the shipyard operation, Seker et al. [22] proposed a risk assessment framework integrated by decision making trial and evaluation laboratory (DEMATEL) and grey system theory which is then applied to a case study in a Turkish shipyard. However, much of the information involved in the shipyard operation risk evaluation is characterized by uncertainty, which may be resolved with fuzzy set theory [23]. 

The causes for accidents and critical risk events in shipyard are mainly attributed to aspects of adverse environments and human-related and/or organizational factors. The environmental factors generally include various conditions on site, such as weather, atmosphere, available hardware or technical equipment in shipyards. Celebi et al. [24] identified the adverse environmental conditions as much as possible by reviewing the operational process in shipyards, and according to the studies implemented by Krstev et al. [25] and Barlas [3], the adverse conditions of materials being toxic, flammable and explosive, dangerous gases, poor ergonomics, and exposure to general hazards may easily trigger the occurrence of shipyard accidents. For instance, Barlas [3] found that the number of fatalities is highest in working temperatures beyond average 25 °C according to the reported shipyard accidents data in Turkey. Tsoukalas and Fragiadakis [1] mapped the relationship between the operational conditions and the occupational risks in the shipyard by multivariable linear regression. The housekeeping at workshops and bad weather conditions were regarded as the two of the main risk factors for shipyard accidents by Barlas and Izci [11]. It is noticeable that the contribution of an adverse environment to shipyard accidents is becoming weaker with the introduction of various advanced technologies into the shipbuilding and repair industry, and the human-related factors are attracting much more attention due to their variability and uncertainty. According to Seker et al. [22], human-related factors were mainly represented in the aspects of individual competence, sociological, psychological and physiological. Based on the investigation of shipyard accidents statistically, Barlas and Izci [11] found that human-related factors were the main reasons for shipyard accidents, such as low education and training, being tired and sleepy, overtime and so on, and similar conclusions were also obtained by Efe [2] who ascribed the main reason for falling from a height accidents to the absence of a safety belt or seat belt during operation. In addition, organizational factors are also important to embed the precautions obtained from accidents analysis into the safety management system [26,27]. The study implemented by Crispim et al. [12] also indicated that the main factors leading to risk events in military shipbuilding are related to humans and organization. 

However, human-related factors involved in shipyard accidents have not been investigated systematically. Even though the importance of human-related factors contributing to shipyard accidents is recognized by many scholars, such as Barlas and Izci [11], Efe [2] and Crispim et al. [12], the studies associated with influencing pathways and mechanisms of human-related factors on shipyard accidents are rare in the existing literature. The reasons may be explained by the complexity and uncertainty involved in the human-related operations in shipyard. There are many stakeholders involved in a shipyard operation, especially for ship repair activities, such as the crew from the ship, workers in the shipyard, ship surveyors, technical engineers from suppliers, all of whom are interrelated together temporarily to implement ship repair work. In addition, it is difficult to monitor effectively the behaviors of humans involved in the shipyard operation. Although the study for human-related factors involved in shipyard operations is still underway, similar studies in other industries have been well developed, such as maritime accidents analysis [28], aviation accidents investigation [29], risk evaluation in process industry [30] and coal mine accidents analysis (Liu et al., 2018). According to the review by Qiao et al. [31], the human-related factor analysis models can be divided into four types, in which various technologies, quantitative or qualitative, are involved. It is noticeable that the qualitative approaches or models prove to be critical to provide an analysis framework, such as the human factors analysis and classification system (HFACS) [32], system theoretic process analysis (STPA) [33], human error assessment and reduction technique (HEART) [34], cognitive reliability and error analysis methods (CREAMs) [35], and system theoretic accident model and process (STAMP) [36]. Based on the qualitative analysis framework, it is necessary to apply quantitative assessment technologies to analyze human-related factors for accident prevention. One of the popular models is the probabilistic-based Bayesian inference, which is widely applied in various risk scenarios. In practice, the BN is frequently integrated with other risk analysis approaches, such as fault tree analysis (FTA) [37], HFACS [31], the success likelihood index method (SLIM) [38] and the technique for order of preference by similarity to an ideal solution (TOPSIS) [39]. The simulation based on information communication technology can also be referred to manage human-related factors [40]. In addition, the technologies associated with artificial intelligence and big data are being applied to assess the human-related factors involved in accidents, such as the artificial neural network (ANN) [28], machine learning [41,42] and data mining [43]. In the present study, the human-related factors involved in shipyard accidents are characterized by obvious uncertainty and ambiguity which can be reasonably handled by fuzzy theory [44]. In addition, there is no doubt that different human-related factors are interrelated logically and, finally, accidents may be triggered. Therefore, it is critical to analyze the relationships among different human-related factors quantitatively, for instance, from the perspective of probability. The principle of FTA is well suitable to represent these logic relationships, and it is possible to determine the probability for every human-related factor (known as an event in the fault tree) [45], which can be used in the following Bayesian inference.

### 1.2. Contribution of This Study

The focus of this study is to establish a double-nested accident analysis and risk assessment framework, under which the fuzzy algorithm and information theory are integrated to quantify and address the uncertainty of human factors and their dependency in shipyard accidents. Moreover, an accident that occurred in a Chinese shipyard is used as a case to verify the proposed methodology. Based on a modified framework of HFACS, the combination of FTA and a fuzzy extent Analytic Hierarchy Process (AHP) is utilized to systematically establish the causal relationship of accidents and address the randomness of risk factors in this study. In addition, BN is adopted to predict the probability of risk because of its powerful learning and reasoning capability. In particular, the introduction of canonical probabilistic models reduces the difficulty of obtaining the conditional probability among nodes in the Bayesian network, and it can deal with the complexity involved in human factors analysis. The model developed in this study can help to address human-related risks in shipbuilding and repair projects from a system perspective, and reduce the incidence of occupational accidents and injuries. The salient features of the proposed methodology are listed as follows:

- A double-nested model for the assessment of human-related risks is proposed by mapping a fuzzy fault tree into a Bayesian network based on HFACS.
- The difficulty of obtaining the conditional probability table of Bayesian network is overcome by canonical probabilistic models.
- Mutual information is introduced to identify main paths of risk propagation.

### 1.3. Organization

The rest of this paper is organized as follows: Section 2 outlines an overview of the research methodology, and the employed models and techniques. The process of establishing the double-nested assessment model and applying it to a case study is presented in Section 3. The discussion and conclusions are presented in Section 4 and Section 5.

## 2. Methodology

**Figure 2:**
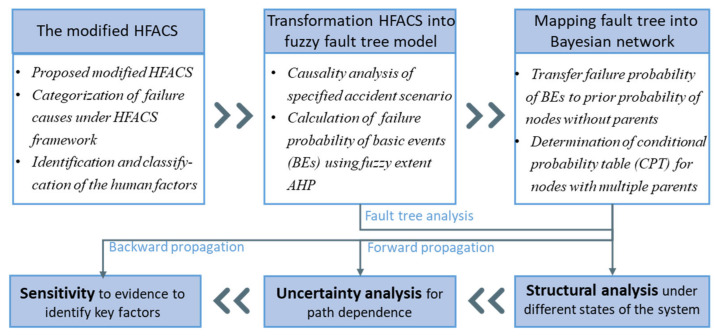
Overview of the proposed methodology.

A complete accident analysis should not only investigate the direct cause of an accident but also identify the deep-seated human risk factors hidden in the system to fundamentally eliminate the latent hazards. For this purpose, the fuzzy fault tree model, which is nested with the hierarchical framework of the HFACS, is first proposed to thoroughly analyze the human factors that contribute to occupational accidents in the shipbuilding and repair industry. Then, it is mapped into BN, which is the double-nested model proposed in this paper, to further explore the characteristics and mechanisms of human factors quantitatively through probabilistic reasoning and uncertainty and sensitivity analysis. The specific schematic diagram is shown in Figure 2.

### 2.1. The Modified HFACS

**Figure 3:**
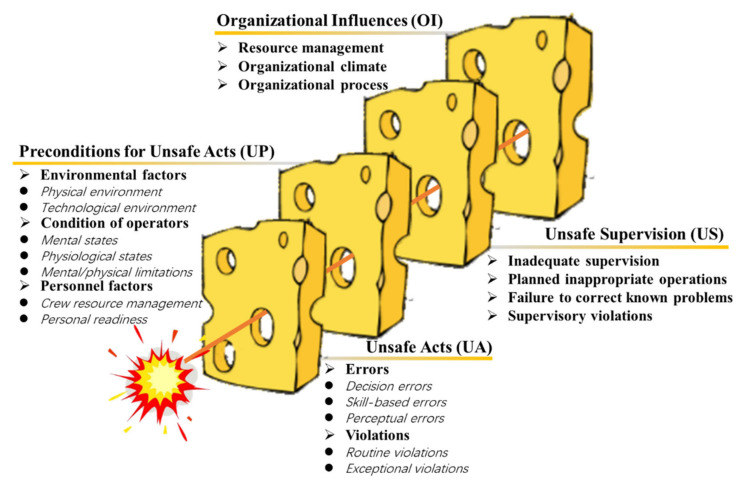
The original framework of HFACS.

HFACS was initially proposed based on the ‘Swiss cheese’ model to analyze the human errors that led to the repeated occurrence of naval aviation accidents in a systematic manner [32], and it has been widely used to analyze the increasing problems of human behavior. Four layers of barriers are included in the ‘Swiss cheese’ model, and active or latent failures lead to holes in different safety barriers, resulting in four types of failures: unsafe acts, preconditions for unsafe acts, unsafe supervision, and organizational influences. An accident occurs when the defense-in-depth barrier system fails. However, the model does not clearly explain what the holes in the cheese slices represent. Accordingly, the HFACS was developed to describe more specific causal categories at each failure layer [46], as shown in Figure 3, to effectively identify the explicit and implicit causes of accidents, thus becoming a comprehensive human factor analysis tool.

HFACS is frequently applied to investigate and analyze the causes of accidents in various industries. Overall, most categories of the original HFACS framework are retained, while some minor optimizations are implemented to better meet the needs of a particular research field [47]. The modification of the HFACS is also implemented in the present study, which is helpful to find the recurring patterns of deficiencies in the safety production system of ship maintenance. A brief description of the improvements follows, and a more detailed illustration of the modified HFACS framework will be discussed in Section 3.2.1.

- In the category of ‘preconditions for unsafe acts (UP)’, one of its subcategories, personnel factors, including personal readiness and crew resource management, has been removed. The ‘crew resource management’ which refers to a series of communication and team cooperation issues that affect personal performance is considered under ‘resource management’ because it is closer to the concept of human resource management, and ‘personal readiness’ is integrated into the ‘conditions of operators’ for discussion.
- In the category of ‘unsafe supervision (US)’, ‘failure to correct known problems’, is related to the situation in which vulnerabilities related to personnel, equipment, etc., are known by supervisors and allowed to continue uncorrected. It is replaced by ‘inadequate process supervision in emergency management’. In most cases, the meaning of ‘failure to correct known problems’ may overlap and cross with the content of ‘inadequate supervision’ and ‘supervision violations’ because there is no restrictive characterization of it.

### 2.2. Fuzzy Fault Tree

#### 2.2.1. Fuzzy Extent AHP

Since it is difficult to obtain quantitative and complete data on human behavior due to its subjectivity and randomness, the quantification of human risk factors often depends on expert judgment. The AHP proposed by Saaty [48] is widely accepted as a concise method and is usually employed to quantify the qualitative indicators based on expert opinions. Based on the hierarchical structure of AHP, the weight or priority of risk factors contributing to the defined scenario can be calculated by pairwise comparison at different levels. However, the crisp value is regarded as inaccurate due to the uncertainty and ambiguity of expert evaluation [49]. Therefore, it is necessary to extend the AHP under fuzzy logic. Chang [50] improved the AHP by combining it with the fuzzy extent analysis method, taking expert knowledge, subjective bias, and unquantifiable and incomplete information into account in the decision-making process. Based on the established hierarchical structure, the fuzzy AHP is able to decompose a complex problem into several simple decision attributes, which are easier to understand and evaluate [51]. In addition, the decision-maker does not need to specify crisp scales for each indicator [52]. Moreover, for the results of pairwise comparison, fuzzy extent AHP can directly obtain the weight vector of indicators at each layer without defuzzification [53]. In this study, fuzzy extent AHP is employed to calculate the weights of human factors, and the fuzzy-valued weight will ultimately be converted into a failure probability for further risk assessment.

#### 2.2.2. Fault Tree Analysis

Fault Tree Analysis (FTA) is one of the classical methods for accident analysis in complex systems. Taking the system failure (i.e., that an accident occurs) as a premise, probable causes can be deductively determined and divided into branch or tip nodes of the fault tree model. The nodes that cannot be decomposed further are basic events (BEs), and the others are intermediate events (IEs). Top event (TE) in the fault tree model refers to the specified accident scenario. Consideration of the logical correlation among events, different logic gates are introduced to express the diverse properties of causal relationships in accidents, and the cause–consequence relationship can be visualized as a tree graph. According to FTA, not only will the direct causes of accidents be investigated, but all the accident causes, including potential risks, will also be found [54]. Therefore, the risk analysis is founded on a reliable basis.

In the qualitative part of FTA, the Boolean algebra reduction method is utilized to determine the minimal cut sets (MCSs) and minimal path sets (MPSs). MCSs represent accident modes, and the greater the number of MCS, the more dangerous the system is. Conversely, MPSs reflect the safe performance of the system. For quantitative FTA, the first step is to calculate the failure probability of BEs, and then, the failure probability of TE can be computed for risk assessment. In the present study, FTA is developed by converting the human factors identified under the HFACS framework into corresponding elements of the fault tree and is mainly utilized for in-depth causality analysis of accidents and identification of MCSs and MPSs.

### 2.3. Bayesian Network

The Bayesian Network (BN) is a multi-element graphical network based on probability and uncertainty. It has been widely used in risk analysis field because of advantages in knowledge representation, autonomous learning, inference and prediction for incomplete information [55]. A given Bayesian network can be expressed as $B=\langle G\langle V,A\rangle ,P\rangle$, where G represents the structure of the BN, which is a directed acyclic graph composed of nodes and their connecting arcs; V is a set of nodes in G, which represents a group of random discrete variables $V=\left\{V_{1},V_{2},V_{3},\cdots ,V_{i}\right\}$, A refers to directional connecting lines, and the internal causality between risk factors is represented by a line pointing from a parent node to a child node. In addition, P is a network parameter that maps the relationship types and strength of the connections between variables with a conditional probability table (CPT). Giving a set of nodes $Z=\left\{V_{1},\cdots ,V_{i}\right\}$, $pa(V_{i})$ is the set of parent nodes for $V_{i}$, the joint probability distribution over Z is expressed as:

(1) $$P(Z)=\prod_{i=1}^{n}P(\left.V_{i}\right|pa(V_{i}))$$

The most prominent function of a BN is diagnostic reasoning. Based on Bayes’ theorem, the prior probabilities of a query set Q are updated to a posterior probability distribution $P(Z\left|E\right.)$, as represented in Equation (2), by inserting variables E, called *evidence*. In general, the query set could be a combination of variables or contain just one variable. In addition, evidence is usually given in the form of accidents, incidents, near misses, etc. [47].

(2) $$P(Z\left|E\right.)=\frac{P\left(Z,E\right)}{P\left(E\right)}=\frac{P\left(Z,E\right)}{\sum {}_{Z}P\left(Z,E\right)}$$

The main issue to implement inferences with BN is the acquisition of the CPT. The conventional methods are observed as sample data learning and expert knowledge [56]. However, for a BN composed of binary discrete variables, the complexity of the joint probability distribution increases exponentially with the increase of composed nodes. That is, if a child node has n parent nodes, then the corresponding CPT of the child node is numbered by $2^{n}$. Faced with such a large data demand, it is difficult for experts to determine $2^{n}$ probability distributions; in addition, sample data are usually not easy to obtain. Therefore, the introduction of canonical models could be an ingenious way to make the training of the probability model easier [57].

#### 2.3.1. Noisy-OR Gate

The Noisy-OR gates are frequently used in BN modeling to calculate conditional probability [58]. It is assumed that the consequence is affected by each cause independently, Noisy-OR gates are usually used to describe the cause-consequence interaction between variables of $X_{1},X_{2},X_{3},\cdots ,X_{n}$ and their child node Y. In particular, Noisy-OR gates require all variables to be binary, that is to say, each variable has two states, which are set as *True* and *False* in this study. In addition, each variable $X_{i}$ has a probability high enough to cause result Y when the other variables are false. Then, the probability of Y under the condition of $X_{i}$ can be mathematically expressed as:

(3) $$P_{i}=P\left(Y\left|{\bar{X}}_{1},\right.{\bar{X}}_{2},\cdots ,X_{i},\cdots ,{\bar{X}}_{n-1},{\bar{X}}_{n}\right)$$

where $P_{i}$ denotes the probability that Y is *True* and ${\bar{X}}_{i}$ represents that the state of $X_{i}$ is *False*.

The next step is to calculate the prior probability of each parent node, then the condition parameters between the parent nodes $X_{1},X_{2},X_{3},\cdots ,X_{n}$ and the child node Y can be approximately determined by:

(4) $$P\left(Y\left|X\right.\right)=1-\prod_{X_{i}\in X}\left(1-P_{i}\right)$$

#### 2.3.2. Leaky Noisy-OR Gate

A leaky node, denoted by $X_{L}$ is added to the original Noisy-OR model to develop the leaky Noisy-OR gate [59], which contains all the potential faults and measurement errors. It is suitable for addressing the situation in which an established BN could not capture all the contributing factors of Y [56]. With the assumption that all the parent nodes are functioning normally, the child nodes may also be in the *False* state due to the existence of leaky nodes. The leaky probability l is defined as the probability that Y is *True* but all of its causes are *False*. In addition, the probability that the state of Y is *True* can be obtained by:

(5) $$P\left(Y\left|X\right.\right)=1-\left[\left(1-l\right)\prod_{X_{i}\in X}\left(1-P_{i}\right)\right]$$

## 3. Application of the Methodology

### 3.1. Case Study: A Suffocation Accident in Shipbuilding

The selected accident case occurred at 3:06 p.m. on 25 May 2019. A cargo ship *Jinhaixiang* was in the dock for maintenance. The third officer opened the valve of the carbon dioxide cylinder negligently, resulting in carbon dioxide in the cylinder entering the collecting pipeline. In the process of emergency response, he grasped the pressure handle on the booster valve by mistake, which caused the carbon dioxide in the collection pipeline to enter the drive pipeline. The cylinder head valve for 84 carbon dioxide cylinders and the main valve leading to the engine room were opened, and a large amount of carbon dioxide was discharged into the engine room in an instant. Due to the lack of unified command and coordination on site, the rescue was chaotic, and the deck ladder was blocked many times, which prolonged the time, as a result, the maintenance personnel and rescue personnel were trapped in the engine room. In addition, most of the personnel did not wear effective protective equipment for rescue, which expanded the number of people who died or were injured due to suffocation. As a result, there were a total of 10 deaths and 19 injuries of on-site maintenance personnel and crew in this suffocation accident.

### 3.2. Human Factor Identification and Classification Based on HFACS

#### 3.2.1. Categorization of Failure Causes

**Figure 4:**
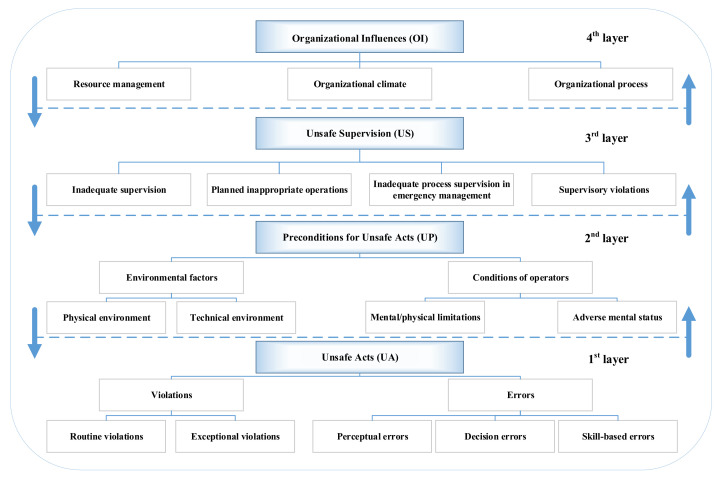
The hierarchy and categories involved in the modified HFACS.

Within the framework of modified HFACS, human risk factors are identified and divided into four layers and 11 categories; in addition, there are five subcategories under ‘Unsafe acts’ and four subcategories within ‘Preconditions for unsafe acts’, as shown in Figure 4. The four levels are continuous in nature, and at each level, a failure or defect can either be explicit, directly affecting the accident, or it can be implicit, temporally removed from the accident but showing no direct impact [60]. Explicit and implicit causes of accidents can be distinguished under the HFACS framework according to Heinrich’s domino theory [61]. It is noticeable that the potential risks at each level may also result in an accident. 

‘Unsafe acts (UA)’ are at the bottom of the HFACS framework, which are the active factors and the most direct causes of accidents. According to ‘The Classification Standard for Casualty Accidents of Enterprise Employees’ [62], these include a series of dangerous practices called errors during the process of workers’ professional activities, such as underestimation of situations, misoperation, and ignorance of warnings. In addition, it also includes abnormal behaviors contrary to the purpose of behavior or the rules of the organization, which are called violations.

The second layer is ‘Preconditions for unsafe acts (UP)’ containing two categories: conditions of operators and environmental factors. According to the domino theory, unsafe acts of human and unsafe states of machinery are the direct causes of accidents [61]. The environmental factors are derived from the expansion of unsafe states of machinery in the modified HFACS, so they are also considered as a direct cause [63]. While the conditions of operators always lead to the unsafe acts mentioned above, they are considered to be indirect causes. As shown in Figure 4, this category consists of inherent defects, such as recklessness, stubbornness, and overreaction, and acquired defects, such as lacking the knowledge and skills of safe production, as well as physical deficiencies [64].

‘Unsafe supervision (US)’ is the indirect cause of accidents at the management level, which consists of inadequate supervision, planned inappropriate operation, inadequate process supervision in emergency response, and supervisory violations. Inadequate supervision reflects the extent to which supervisors perform safety management responsibilities, including the supervision and management of personnel and resources. Planned inappropriate operation refers to the management and assignment of work, including risk management, operator matching, and setting work schedules. Inadequate process supervision in emergency response is especially concerned with the timely prevention and correction of unsafe behaviors in the process of emergency response. In addition, supervisory violations are generally focused on the on-site management.

‘Organizational influences (OI)’ are also the hidden causes that lead to accidents. Such defects may not have a direct impact, but they are likely to cause hazards under the combined effect of other factors. This category consists of three types: resource management, the organizational climate, and the organizational process. In this study, resource management takes the perspective of human resource management. The organizational climate is an invisible risk factor that has a subtle impact on human actions. In addition, the organizational process is the response to the operation of the safety management system, and it can test the stability of the system.

#### 3.2.2. Human Factor Identification and Classification

**Figure 5:**
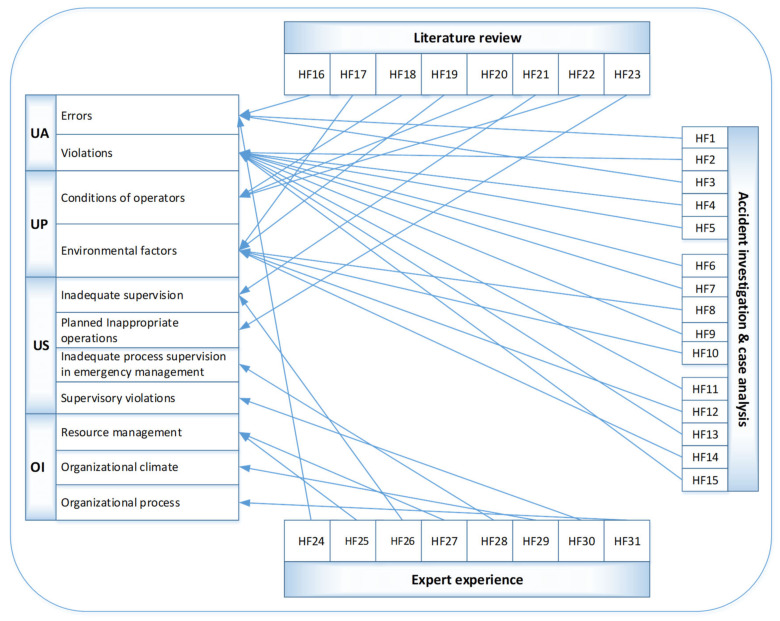
Process for human factor identification and classification.

In this section, the proposed HFACS is used to analyze the explicit and implicit causes leading to the occurrence of the example accident used by identifying human factors at each layer and in each category. To identify all human factors in the accident scenario described, expert experience, a literature review, accident investigation and case analysis are combined and applied. After safety meetings, a questionnaire survey, and literature review, the risk elements associated with human factors are summarized in Table 1. The detailed identification and classification process is shown in Figure 5. 

As proposed in Section 2.1, we have developed a human factor identification and classification framework characterized by a hierarchical structure. Under this framework, the contributory factors mentioned in the accident investigation report are initially extracted as the initial risk database. Then, a literature analysis is used to handle the incomplete and unclear information in the report.

In addition, expert knowledge is introduced as an important supplement. One way is to supplement and adjust human factors by obtaining this knowledge through questionnaire surveys and interviews to improve the comprehensiveness and representativeness of the identified elements. Furthermore, by organizing symposiums, the scientific and rational classification of risk factors will be unanimously recognized by experts. 31 human factors were ultimately identified and divided into four groups: unsafe acts (*n* = 13), preconditions for unsafe acts (*n* = 9), unsafe supervision (*n* = 5) and organizational influences (*n* = 4). All human-related risk factors and their symbols and descriptions are shown in Table 2.

### 3.3. Fuzzy Fault Tree Modeling

**Figure 6:**
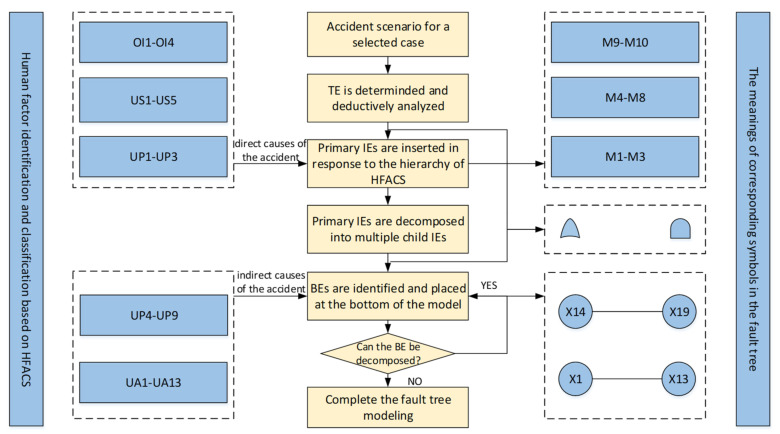
The procedure of fault tree modeling based on HFACS.

In this section, all identified human factors will be transformed into elements of the fault tree model. As mentioned, the direct causes of accidents are aggregated into two categories, namely, ‘Unsafe acts’ and ‘Environmental factors’ in the modified HFACS, which are illustrated as BEs in the fault tree. In addition, indirect causes are transferred to corresponding IEs, which connect the TE and BEs in the fault tree. The process for converting the HFACS to a fault tree is presented in Figure 6. UA1–UA13, UP4–UP9, UP1–UP3, US1–US5, and OI1–OI4 in HFACS correspond to X1–X13, X14–X19, M1–M3, M4–M8, and M9–M10, respectively.

#### 3.3.1. Causality Analysis of the Accident Scenario

Figure 6 shows the process of fault tree modeling based on the HFACS architecture. The static fault tree model is built starting with the determination of the TE ‘suffocation accident’.

As the first step, the vertical relationship between IEs and BEs is determined according to causal analysis. Deductive analysis is carried out from the TE to BEs, and the causes of the accident are separated layer by layer. It should be noted that in response to the hierarchical progression principle of the HFACS (represented by the directed lines in Figure 4), ‘Organizational influences’ are converted to primary intermediate events directed to the top event in the fault tree. In other words, the primary intermediate events can be further decomposed into multiple child intermediate events. Subsequently, ‘Unsafe supervision’ and ‘Conditions of operators’ are inserted as child IEs through cause-effect analysis.

**Figure 7:**
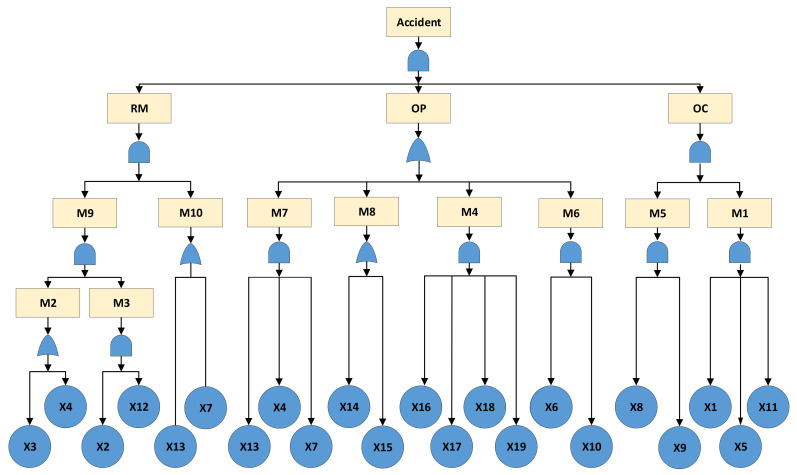
Cause-effect diagram of the fault tree for the suffocation accident.

In the second step, logic gates are selected to connect events at different levels to complete the cause-consequence fault tree modeling. Logical AND and OR gates are the most common Boolean connectors; AND indicates that multiple events fail at the same time to cause the system failure, and OR means that any event may lead to an accident. Through causality analysis of the accident, ‘Resource management’ (RM), ‘Organizational climate’ (OC) and ‘Organizational process’ (OP) all have the possibility of leading to the TE, so OR gates are set between the TE and the three primary IEs. By analogy to BEs with the same method, a causality diagram of the fault tree is constructed, as shown in Figure 7, which includes 13 IEs, 19 BEs, 10 OR gates, and 4 AND gates. The meanings of the corresponding symbols are given in Figure 6.

#### 3.3.2. Probability Calculation of BEs Using the Fuzzy Extent AHP

The quantitative analysis of the FTA would be conducted in the present section. The fuzzy extended AHP is introduced to compute the failure possibilities of BEs. Five non-homogenous experts are firstly invited to make independent judgments on the contribution of risk factors, and triangular fuzzy numbers are used to make judgments intuitively. The basic information for these five experts can be found in Table 1. The definition and operational principles of triangular fuzzy numbers are as follows [50]:

Let $\tilde{N}$ be a triangular fuzzy number defined on R such that its membership function $\mu_{N}(x):R\to \left[0,1\right]$ is equal to:

(6) $$\mu_{N}\left(x\right)=\left\{\begin{array}{cc} \frac{x}{m-l}-\frac{l}{m-l} & ,\;x\in \left[l,m\right]\\ 0 & ,\;\mathrm{otherwise}\\ \frac{x}{m-u}-\frac{u}{m-u} & ,\;x\in \left[m,u\right] \end{array}\right.$$

where l≤m≤u, m is the modal value of the fuzzy number, and l and u stand for the lower and upper bounds of $\tilde{N}$, respectively. The triangular fuzzy number is denoted as $\tilde{N}=(l,m,u)$, and when u−l=0, the judgment is not ambiguous.

Consider two triangular fuzzy numbers ${\tilde{N}}_{1}=(l_{1},m_{1},u_{1})$ and ${\tilde{N}}_{2}=(l_{2},m_{2},u_{2})$; their extent operational laws are defined as follows:

(7) $${\tilde{N}}_{1}⊕{\tilde{N}}_{2}=(l_{1}+l_{2},m_{1}+m_{2},u_{1}+u_{2})$$

(8) $${\tilde{N}}_{1}⊗{\tilde{N}}_{2}=(l_{1}l_{2},m_{1}m_{2},u_{1}u_{2})$$

(9) $$\lambda \tilde{N}=(\lambda l,\lambda m,\lambda u)$$

(10) $${\tilde{N}}^{-1}=(\frac{1}{u},\frac{1}{m},\frac{1}{l})$$

Then, the fuzzy extent AHP is introduced to obtain the failure rate of human factors. Expert credibility has been added to the process to reduce both empirical limitations and subjective bias. In practice, because of the comprehensive effect of the personal knowledge background, work experience and so on, the reference weights of expert opinions on a certain issue are not equal. Nevertheless, whether the expert evaluation is scientific or not, it has a direct impact on the results of the study. Professional position, length of service, education level and job title [54].

As shown in Table 3, the authority of each expert in a professional academic or practical field is considered according to their knowledge and skills, which are accumulated through different work experiences. In addition, each aspect is divided into diverse scoring standards. According to Equations (11) and (12), expert credibility can be calculated.

Let $E=\left\{e_{1},\right.\left.e_{2},\cdots ,e_{n}\right\}$ be the expert reliability set; the expert comprehensive reliability value $e_{k}$ is expressed as:

(11) $$e_{k}=e_{PPk}+e_{LEk}+e_{ELk}+e_{JTk}$$

With n experts, the credibility of each expert $\lambda_{e}$ is defined as:

(12) $$\lambda_{e}=\frac{e_{k}}{\sum_{k=1}^{n}e_{k}},\;\;\;\lambda_{k}>0,\;k=1,2,\cdots ,\;n\;\;and\sum_{k=1}^{n}\lambda_{k}=1$$

The algorithm of the fuzzy extent AHP is as follows:

Step 1: Development of fuzzy reciprocal judgment matrix. Set ${\tilde{N}}^{K}(K=1,2,\ldots ,k)$ as the judgment opinion of expert K. Through a pairwise contribution comparison of factors at each level, the triangular fuzzy judgment matrix $N^{K}$ is developed as:

(13) NK=N˜ijK=1N˜12K⋯N˜1nK1/N˜12K1⋯N˜2nK⋮⋮⋱⋮1/N˜1nK1/N˜2nK 1        ∀i,j=1,2,…,n

where i is the number of rows of the indicators, j is the number of columns, and ${\tilde{N}}_{ij}^{K}$ is the comparison value given by Expert K, which is a triangular fuzzy number.

Step 2: Determination of the fuzzy synthetic extent value. Applying the extent analysis method, the comprehensive fuzzy value $D_{i}$ of the ith row element relative to the other row elements, i.e., the initial weight, is calculated as follows:

(14) $$D_{i}={\sum_{j=1}^{n}{\tilde{N}}_{ij}⊗\left[\sum_{i=1}^{n}\sum_{j=1}^{n}{\tilde{N}}_{ij}\right]}^{-1}$$

To obtain $\sum_{j=1}^{n}{\tilde{N}}_{ij}$, perform the ⊗ operation on n extent analysis values for a specific matrix such that,

(15) $$\sum_{j=1}^{n}{\tilde{N}}_{ij}=\left(\sum_{j=1}^{n}l_{ij},\sum_{j=1}^{n}m_{ij},\sum_{j=1}^{n}u_{ij}\right)$$

(16) $$\sum_{i=1}^{n}\sum_{j=1}^{n}{\tilde{N}}_{ij}=\left(\sum_{i=1}^{n}\sum_{j=1}^{n}l_{ij},\sum_{i=1}^{n}\sum_{j=1}^{n}m_{ij},\sum_{i=1}^{n}\sum_{j=1}^{n}u_{ij}\right)$$

Step 3: Apply the fuzzy number comparison rule, and calculate the degree of possibility of ${\tilde{N}}_{1}=\left(l_{1},m_{1},u_{1}\right)\geq {\tilde{N}}_{2}=\left(l_{2},m_{2},u_{2}\right)$, which is found as:

(17) $$V\left({\tilde{N}}_{1}\geq {\tilde{N}}_{2}\right)=\sup_{x\geq y}[\min(\mu_{N_{1}}\left(x\right),\mu_{N_{2}}\left(y\right))]$$

(18) $$V\left({\tilde{N}}_{1}\geq {\tilde{N}}_{2}\right)=\mathrm{hgt}\left({\tilde{N}}_{1}\cap {\tilde{N}}_{2}\right)=\left\{\begin{array}{cc} 1 & ,\;m_{1}\geq m_{2}\\ \frac{l_{1}-u_{2}}{\left(m_{2}-u_{2}\right)-\left(m_{1}-l_{1}\right)} & ,\;m_{1}\leq m_{2},u_{1}\geq l_{2}\\ 1 & ,\;otherwise \end{array}\right.$$

To compare ${\tilde{N}}_{1}\geq {\tilde{N}}_{2}$, the values $V\left({\tilde{N}}_{1}\geq {\tilde{N}}_{2}\right)$ and $V\left({\tilde{N}}_{2}\geq {\tilde{N}}_{1}\right)$ are required. The degree of possibility for a convex triangular fuzzy number being greater than k convex triangular fuzzy numbers ${\tilde{N}}_{i}\left(i=1,2,\ldots ,k\right)$ can be defined as:

(19) $$V\left(\tilde{N}\geq {\tilde{N}}_{1},{\tilde{N}}_{2},\ldots ,{\tilde{N}}_{i}\right)=\min V\left(\tilde{N}\geq {\tilde{N}}_{i}\right)\;\;i=1,2,\ldots ,k$$

Step 4: By standardizing the above weights, the standard values SV of each indicator can be obtained as:

(20) $$SV={\left(\min V\left({\tilde{N}}_{1}\geq {\tilde{N}}_{k}\right),\min V\left({\tilde{N}}_{2}\geq {\tilde{N}}_{k}\right),\ldots ,V\left({\tilde{N}}_{n}\geq {\tilde{N}}_{k}\right)\right)}^{T}\;\;k=1,2,\ldots ,n$$

Step 5: Calculate the integrated values IV of each indicator. The integrated value of the ith indicator in the K layer is obtained by:

(21) $$IV_{i}=\prod_{K-n}^{K}SV_{i}^{K}\;\;\;\;K=1,2,\cdots ,n$$

### 3.4. Mapping the Fuzzy Fault Tree into the Bayesian Network

To map the fuzzy fault tree into BN, the transformation relationship between the corresponding components needs to be established. According to the logical relationship expressed by fuzzy fault tree and its representation in BN, the transformation from fuzzy fault tree to BN is presented as follows:

- All BEs in the fuzzy fault tree are considered as parent nodes in the BN, and all IEs are regarded as child nodes.
- The nodes in the BN are connected according to the relationship between various layers of events in the fuzzy fault tree, and the direction of edges is consistent with the cause–consequence relationship in the fuzzy fault tree.
- The failure probability of each BE is directly assigned to the corresponding parent node in the BN as its prior probability distribution.
- The conditional probabilities of each variable, i.e., the CPT, provide the functional extension of logic gates in the fuzzy fault tree.

#### 3.4.1. Prior Probabilities for Nodes without Parents

As mentioned before, the prior probability distribution for nodes without parents also corresponds to the failure probability of BEs. The fuzzy extent AHP in Section 3.3.2 is carried out as the first step. Five experts are employed to evaluate the contributing factors of the accident. Their backgrounds are illustrated in Table 4. According to the scoring standard in Table 3, expert credibility is calculated as shown in Table 4. The credibility of the experts are unequal in the heterogeneous decision-making group.

Then, by filling out the questionnaire of indicator evaluation, five experts made their pairwise judgment results on the failure possibility of human factors at different levels. Fuzzy evaluation given by the expert group is shown in Appendix A (Table A1, Table A2, Table A3 and Table A4). Each risk factor that may lead to the occurrence of accidents is compared in pairs utilizing Equations (13)–(21). In addition, the triangular fuzzy scale is applied in the present study as a reference for converting expert fuzzy judgment terms to values [66]. To illustrate the calculation process more specifically, the indicators of ‘Errors’ (X1–X4) are taken as an example. A fuzzy judgment matrix is developed on the basis of the five experts’ judgments. Then, the possibilities of the human factors related to the accident are compared using triangular fuzzy numbers by:

$$d(X_{1})=\min V\left(D_{X_{1}}\geq D_{X_{2}},D_{X_{3}},D_{X_{4}}\right)=\min(1,0.9277,1)=0.9277$$

$$d(X_{2})=\min V\left(D_{X_{2}}\geq D_{X_{1}},D_{X_{3}},D_{X_{4}}\right)=\min(0.9327,0.8661,1)=0.8661$$

$$d(X_{3})=\min V\left(D_{X_{3}}\geq D_{X_{1}},D_{X_{2}},D_{X_{4}}\right)=\min(1,1,1)=1$$

$$d(X_{4})=\min V\left(D_{X_{4}}\geq D_{X_{1}},D_{X_{2}},D_{X_{3}}\right)=\min(0.8967,0.9701,0.8228)=0.8228$$

The above values are standardized and integrated with the influence degree of the upper-level indicators, and the calculation results are as follows:

$$SV={\left(\;0.9277,0.8661,1,0.8228\right)}^{T}=\left(0.2565,0.2395,0.2765,0.2275\right)$$

$$IV=0.3324⊗\left(0.2565,0.2395,0.2765,0.2275\right)=\left(0.0853,0.0796,0.0919,0.0756\right)$$

Ultimately, the probability of each identified human factor is calculated. To convert the impact weight of human factors (crisp failure possibility) into failure probability, the approach proposed by Onisawa [67] is employed to develop a functional relationship between the failure possibility P and the failure probability FP. The conversion algorithm is expressed in Equation (22). The complete arithmetic results are shown in Table 5.

(22) $$FP=\left\{\begin{array}{cc} \frac{1}{10^{K}} & ,\;P\neq 0\\ 0 & ,\;P=0 \end{array}\right.\;\;\;\;\;\;\;\;\;\;,K=2.301\times {\left[\left(\frac{1}{P}-1\right)\right]}^{1/3}$$

#### 3.4.2. Equivalent CPT for Nodes with Multiple Parents

**Figure 8:**
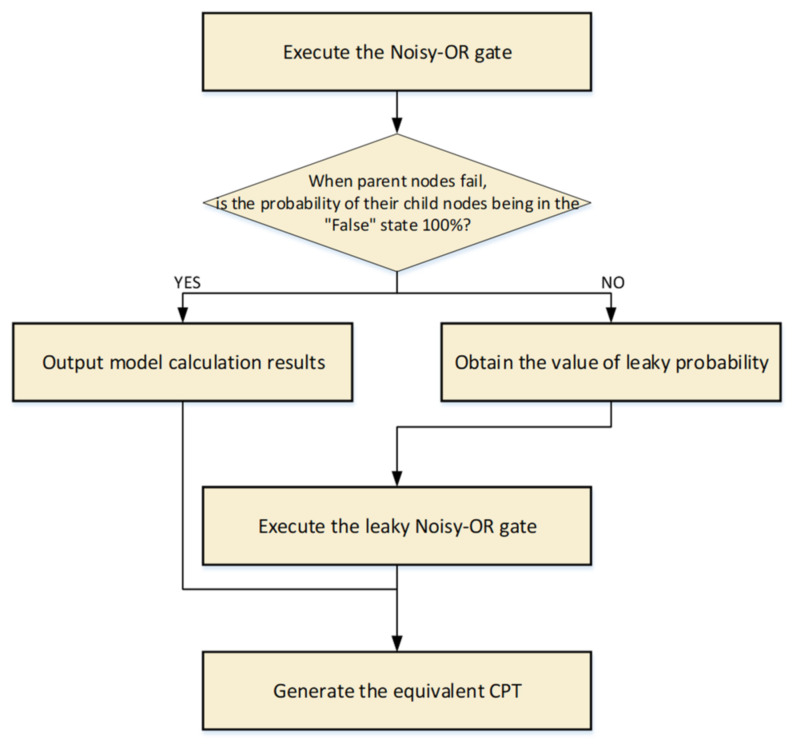
Operational flow chart of the calculation of the equivalent CPT.

For nodes with parent nodes (i.e., child nodes), prior probabilities can be acquired through the forward reasoning function of the BN, in which the calculation of the conditional probability is critical. As mentioned in Section 2.3, the Noisy-OR gate and leaky Noisy-OR gate are the canonical probabilistic models applied in this study. First, a Noisy-OR gate is used to calculate the conditional probability distribution of different node state combinations. According to the conditional function shown in Figure 8, when all parent nodes fail at the same time and the probability of their child nodes being *False* is 100%, the result of the Noisy-OR gate is considered to meet the scientific criteria of the study, and the leaky Noisy-OR gate is no longer used. Meanwhile, the leaky probability l=0. In addition, if the opposite is true, the functional algorithm leaky gate is executed. 

**Figure 9:**
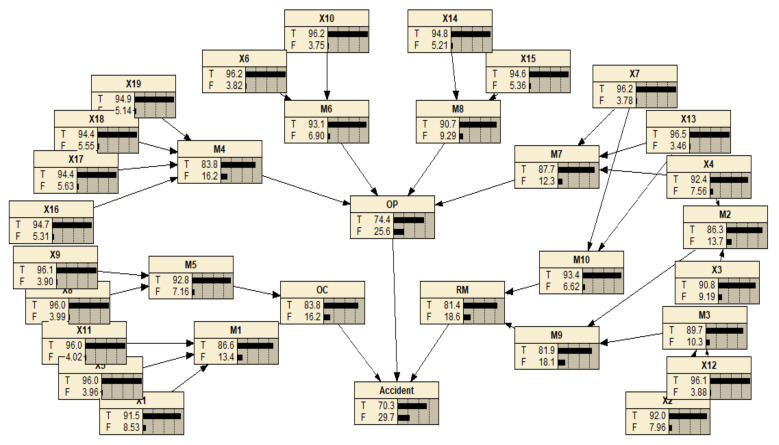
The prior probability distribution of nodes in the developed Bayesian network (BN).

The failure probability of each human factor calculated in Section 3.4.1 is assigned to the prior probability of parent nodes in the BN. According to the operation shown in Figure 8, the equivalent CPT is obtained. The node ‘Risk-seeking with weak security awareness’ (M1) with three parent nodes (X1, X5, X11) is taken as an example. As calculated in Section 3.4.1, $P_{X_{1}}=0.0853,P_{X_{5}}=0.0396,P_{X_{11}}=0.0402$; then, the Noisy-OR gate is utilized to compute the probability distribution under eight different conditions, where $P\left(M_{1}\left|{\bar{X}}_{1},{\bar{X}}_{5},{\bar{X}}_{11}\right.\right)=0.1568\neq 1$. Therefore, the leaky Noisy-OR gate is used to recalculate the conditional probability distribution of this group of nodes. After all conditional probabilities are elicited and entered into Netica, the prior probability of child nodes in the BN could be generated through automatic reasoning, as shown in Figure 9. 

## 4. Results and Discussion

### 4.1. Structural Analysis under Different States of the System

The purpose of structural analysis of static fault tree is to explore the rules of system failure due to common causes. As mentioned in Section 2.2.2, each MCS represents a possible situation in which one or more risk factors cause an accident, while each MPS indicates a condition in which the accident does not occur. By simplifying the fault tree model shown in Figure 7 based on Boolean algebra, MCSs can be obtained; then, its dual success tree is established to calculate the MPSs. Here, FreeFta is used for the determination of MCSs and MPSs, and the results are listed in Table 6. Without considering the failure possibility of risk factors, structural analysis under different states of the system, that is the state of the top event is True or False, respectively, shows that:

- There are 33 MCSs, approximately twice as many as MPSs. Each MCS contains a small number of BEs. Since the MCS with fewer events is more likely to cause the system failure than the MCS with more events, the state of this system is unstable and the probability of accident occurrence is higher.
- Regarding the MCSs that contain only one BE, it can be found that the BEs all belong to the category of ‘Violations’. In addition, ‘Environmental factors’ only appear in the MCSs composed of multiple BEs, that is to say, they cannot lead to system failure independently. Therefore, ‘Violations’ are more likely to cause accidents than ‘Environmental factors’. That is, comparison of the unsafe behavior of humans and the unsafe state of machinery and materials, the former contributes more to accidents.
- There are 17 MPSs, among which four MPSs contain 13 BEs (the largest number of BEs). Generally, the more BEs contained in an MPS, the more complex the technical problems in the system, and more resources are required to ensure the TE does not occur. Therefore, the MPSs consisting of a larger number of BEs are considered to be weak links that easily cause system failure.

### 4.2. Uncertainty Analysis for Path Dependence

Bidirectional reasoning and probabilistic prediction can be realized in a BN by information updates on evidence, which is called evidence propagation [68]. The observed evidence is entered into the model developed in Figure 9 as causes, and updates the probability of all non-evidence nodes, including target variables. Herein, information theory [69] is applied to deal with the uncertainty of random variables, which allows us to model the conditional dependence of child nodes on parent nodes. As a result, the most possible path of the domino effect [70] can be predicted such that the deterioration of dangerous situations may be controlled by cutting off the path dependence of risk propagation.

Considering a discrete random variable $X=\left\{x_{1},\right.\left.x_{2},\cdots ,x_{n}\right\}$, when its value is equal to x, its probability is expressed as the probability mass function P(x). The amount of uncertainty of X with multiple states can be measured using the definition of information entropy as follows:

(23) $$H(X)=-\sum_{i=1}^{n}p\left(x_{i}\right)\log_{b}\left(p\left(x_{i}\right)\right)$$

where $\sum_{x\in X}P\left(x\right)=1$, and the base of logarithm is usually 2 or e. In the present study, it is set to e. The higher the information entropy is, the more statistical information is needed to eliminate the uncertainty of variables.

Considering two random variables X and Y, to measure their dependence, we need to introduce the concept of mutual information, which is expressed as follows:

(24) $$I(X,Y)=H\left(X\right)-H\left(X\left|Y\right.\right)$$

where $H\left(X\left|Y\right.\right)$ is the conditional entropy of X when Y is known, and it can be calculated as follows:

(25) $$\begin{array}{ll} H\left(X\left|Y\right.\right) & =-\sum_{y}p\left(y\right)H\left(X\left|Y=y\right.\right)\\ & =-\sum_{y}p\left(y\right)\sum_{x}p\left(x\left|y\right.\right)\log p\left(x\left|y\right.\right)\\ & =-\sum_{y}\sum_{x}p\left(y\right)*p\left(x\left|y\right.\right)\log p\left(x\left|y\right.\right)\\ & =-\sum_{y}\sum_{x}p\left(x,y\right)\log p\left(x\left|y\right.\right)\\ & =-\sum_{x,y}p\left(x,y\right)\log p\left(x\left|y\right.\right) \end{array}$$

Since conditional entropy measures the uncertainty contained in X when Y is known, mutual information quantifies how much uncertainty of the target variable X is reduced under given evidence conditions. In this study, an important assumption is put forward that under a given condition Y, if the target variable X in non-evidence nodes has the minimum entropy, the dependent path between X and Y is considered to play a leading role in risk propagation. In other words, if mutual information I(X,Y) is the largest, the dependence between the two is the strongest among many factors that affect X. Thus, the primary dependent path in risk chains can be determined using Bayesian reasoning and information theory.

**Figure 10:**
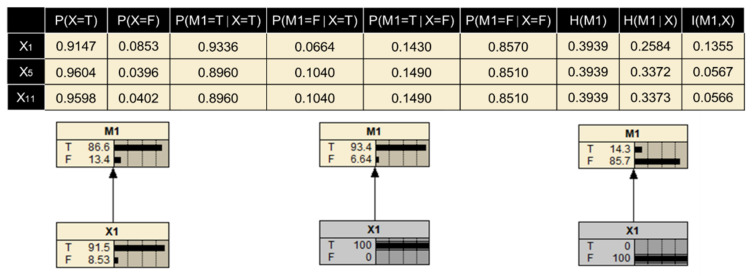
Uncertainty to the target variable under different conditions.

**Figure 11:**
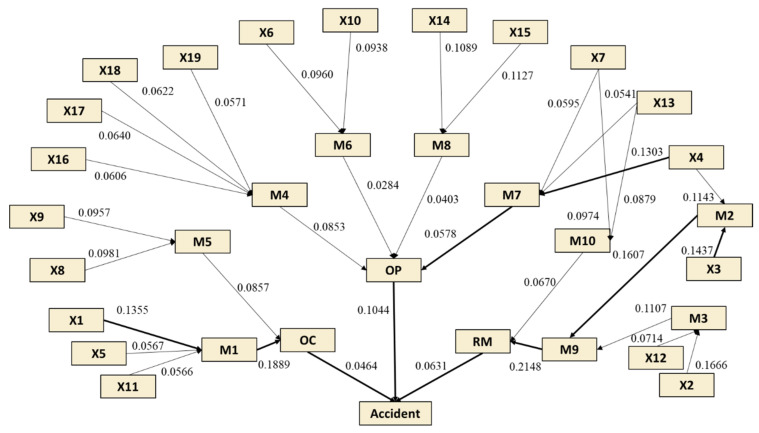
Degree of dependence among risks in different propagation paths.

In this accident scenario, M1 is selected as the target variable, and X1, X5, X11 as the known evidence variables respectively. The corresponding value of entropy and dependence is shown in Figure 10. Similarly, the degree of dependence for the path of all risk propagation can be calculated using Equations (23)–(25), and the results are shown in Figure 11. It is evident that the components in a risk chain related to ‘Resource Management’ (RM) are highly dependent, among which ‘X3→M2→M9→RM→Accident’ is the most significant risk propagation path. The continuous failure of these factors can easily lead to the occurrence of accidents. From the other two aspects of ‘Organizational influences’, the typical risk paths dependence on domino effect are ‘X4→M7→OP→Accident’ and ‘X1→M1→OC→Accident’ respectively. Take the former path as an example, if the operator makes mistakes when taking emergency actions, it would increase the difficulty of emergency response and likely lead to the failure of emergency rescue. At this point, the dangerous situation is irreversible. According to the path of risk dependence, whether the hazard can be mitigated mainly depends on the guidelines in an emergency rescue. The better the emergency management, the less damage it causes and the less likely it is to develop into a serious accident. Therefore, it can provide a reference for taking effective risk mitigation strategies from the perspective of risk transmission, so as to prevent the further expansion of the losses.

### 4.3. Sensitivity to Evidence to Identify Key Factors

The key factors influencing the occurrence of accidents can be inferred through backward propagation of BN with the Pareto principle, which is helpful for the precision management of risk sources. In this study, the sensitivity analysis is incorporated to achieve this purpose. Under specified conditions, the relative importance of the independent variable to the specific dependent variable is calculated by Equation (26). A risk factor with a higher ratio of change (*RoC*) is considered to be more important [30].

(26) $$RoC\left(X_{i}\right)=\frac{\xi \left(X_{i}\right)-\zeta \left(X_{i}\right)}{\zeta \left(X_{i}\right)}$$

**Figure 12:**
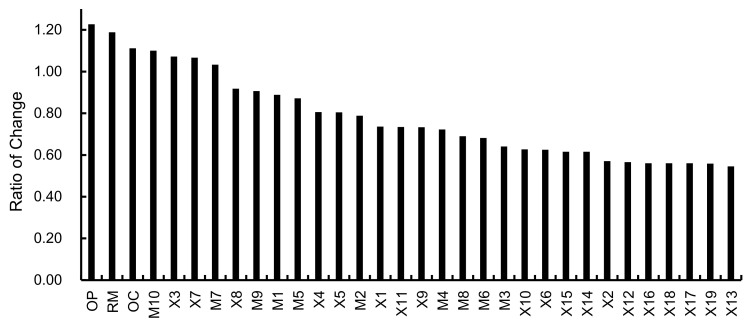
Sensitivity analysis of human factors.

In the developed BN, the failure probabilities of all identified human factors are updated to posterior probabilities by setting the state of ‘Accident’ to *False*. Then, the sensitivity of factors can be quantified according to Equation (26), and the results are shown in Figure 12. The three human factors with the highest sensitivity are ‘Organizational process (OP)’, ‘Resource management (RM)’, and ‘Organizational climate (OC)’, all of which belong to the category of ‘Organizational influences’, and their *RoC* are 1.2266, 1.1882 and 1.1111, respectively. They are considered the main causes of the accident, and OP has the largest contribution among them. Additionally, we set the states of ‘Accident’ and ‘OP’ to *False* and observe the change of their parent nodes, i.e., ‘Lax daily safety management and inspection (M4)’, ‘Unreasonable ship repair plan (M6)’, ‘Inadequate guidelines in emergency rescue (M7)’, and ‘Inadequate on-site control (M8)’. Then, the values of *RoC* for these nodes are calculated as 0.5520, 0.5259, 0.3520, and 0.5350, which reflects the fact that daily safety inspection and supervision are particularly important for accident prevention.

**Figure 13:**
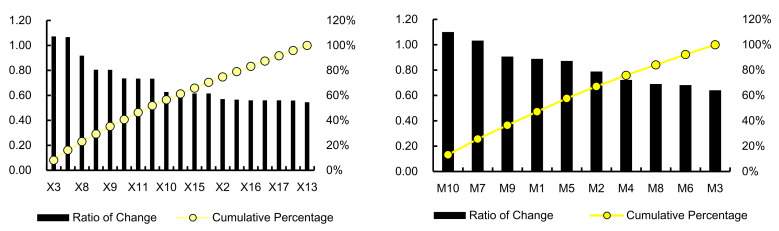
Identification of key human factors involved in accidents.

The three factors with sensitivity slightly less than ‘Organizational influences’ are ‘Lack of team communication and cooperation (M10)’, ‘Operating errors (X3)’, and ‘Rescue without plan (X7)’. This indicates that personnel involved in shipyard operations, such as the operators of engines, electricians, dock repair workers, comprehensive workshop operators, and production supervisors, should pay attention to coordination and cooperation with the members of the organization, and workers should always be careful to avoid mistakes during operation. In addition, we calculate the cumulative percentage of the causes of accidents other than ‘Organizational influence’. As shown in Figure 13, the top 20% of human factors are considered as the key factors leading to the accident according to the Pareto principle [71]. In addition to the three factors (M10, X3, X7) analyzed previously, there are still two key human factors, i.e., ‘Inadequate guidelines in emergency rescue (M7)’ and ‘Entering confined space without assessing air (X8)’. It indicates that the organizational and supervisory ability of managers in the process of emergency management plays an important role in controlling the occurrence of accidents and reducing the number of occupational injuries.

## 5. Conclusions

In this study, a double-nested model that integrates the superiorities of HFACS, fuzzy fault tree and BN is proposed for comprehensive risk assessment and path dependence analysis. The hierarchical characteristic of the HFACS is firstly utilized for potential risk identification and classification. In particular, not only is the classification of risks implemented at different levels of the HFACS but also the risks are divided into direct and indirect causes of accidents. Then, the elements of HFACS are transformed to corresponding BEs and IEs in the fault tree to systematically represent causal associations under specific accident scenarios. A fuzzy fault tree is developed with the application of fuzzy extent AHP to calculate the probabilities of BEs, which is further mapped into a BN to expand the bidirectional propagation function and uncertainty and sensitivity analysis. In addition, an equivalent CPT of nodes with multiple parents in the BN is generated by introducing canonical probabilistic models, namely, the Noisy-OR gate and leaky Noisy-OR gate. Finally, the methodology was applied to a suffocation accident that occurred in a shipyard.

In general, the double-nested model developed in this paper has the functions of perception, inference, and prediction. Through the empirical application of the aforementioned model for a case accident, the safe or failure state of the production system under different combinations of human factors is discussed. It vividly reflects the significant distinction between the combination of risk factors and the sequence of risk failures, which is also one of the functional differences between FFT and BN. After that, the dependence paths between risk factors are analyzed by combining the information theory with the forward propagation function of the BN to demonstrate the regular pattern of risk propagation. In these path dependencies, organizations that are experiencing failure are prone to further failure and the threat of potential occupational accidents. In addition, backward propagation combined with sensitivity analysis is performed to identify key human factors. The output of the model is consistent with the actual situation, and risk factors not investigated in the accident report are also identified and discussed. The research results could provide a reference for further improvement in industrial accident prevention and safety management from two aspects: one is to eliminate the hidden dangers of accidents, and the other is to control the adverse evolution of accidents.

## Figures and Tables

**Table 1:** Source of human factors and their corresponding designation.

Source	Item	Designation	Item	Designation
Accident investigation and case analysis	HF1	Operating errors	HF2	Distracting behavior during work
	HF3	Emergency response failure	HF4	Long duration of work in confined space
	HF5	Improper use of personal protective equipment (PPE) and protective clothing	HF6	Dangerous situation not reported in time
	HF7	Poor risk perception prior to work	HF8	Poor ventilation system
	HF9	Rescue without plan	HF10	Defective device
	HF11	Entering into confined space without assessing the air	HF12	Improper pipeline interconnection
	HF13	Fail to observe entry permit associated with risky confined space	HF14	Failure of safety equipment
	HF15	Poor teamwork		
Literature review	HF16	Underestimation of hazardous situations	HF17	Multiple hazard sources
	HF18	Risk-seeking with weak security awareness	HF19	Limitations of the operating environment
	HF20	Poor competence	HF21	Fail to implement a safety management system and operation procedures
	HF22	Lack of working experience	HF23	Unreasonable ship repair planning
Expert experience	HF24	Delay in emergency response	HF25	Inadequate safety briefing and training
	HF26	Lax daily safety management and inspection	HF27	Lack of team communication and cooperation
	HF28	Inadequate guidelines in emergency rescue	HF29	No positive safety culture and atmosphere
	HF30	Inadequate on-site control	HF31	Safety management system needs to be modified

**Table 2:** (**A**) Identification and description of the human factors under HFACS-Unsafe acts; (**B**) Identification and description of the human factors under HFACS-Preconditions for unsafe acts; (**C**) Identification and description of the human factors under HFACS-Unsafe supervision; (**D**) Identification and description of the human factors under HFACS-Organizational influences.

**(A)**		
**Risk Factor**	**Description**	**Source**
HF16→UA1	Unaware of the possibility of occurrence and the adverse impact of accidents, so that risks are often underestimated and ignored.	[10,65]
HF24→UA2	No immediate measures are taken to deal with the emergency, such as closing the leakage pipeline valve, blocking the leakage point of equipment, or stopping the delivery of gas; therefore, the hazard is not effectively controlled.	Expert experience
HF1→UA3	Misoperation of buttons, valves, wrenches, and handles, or abnormal opening of dangerous devices, resulting in toxic gas leakage.	Accident investigation and case analysis
HF3→UA4	Making mistakes when taking emergency disposal measures, such as improper use of equipment or incorrectly turning the device on or off, resulting in safety device failure.	Accident investigation and case analysis
HF5→UA5	Not applying PPE such as respirators and breathing apparatus, or not properly wearing protective clothing according to regulations, thus operating without protective measures.	Accident investigation and case analysis
HF7→UA6	No prior understanding of the hazards and control countermeasures of operations, and beginning work without an explanation of safety measures.	Accident investigation and case analysis
HF9→UA7	The rescue action lacks unified organization and command and the order is chaotic, for example, because the rescuers do not wear effective protective equipment or the escape route is blocked.	Accident investigation and case analysis
HF11→UA8	The ventilation and diffusion of toxic and harmful gases are not considered and oxygen measurements are not taken before entering confined spaces, which violates the safety operation rules.	Accident investigation and case analysis
HF13→UA9	Risky entry of dangerous environments and confined spaces for operation without permission and approval from a superior.	Accident investigation and case analysis
HF15→UA10	Failure to notify the on-duty personnel of the entry plan, thus performing a separate operation without monitoring measures and having no reliable communication with external parties.	Accident investigation and case analysis
HF2→UA11	Attention is not completely focused on the work during operations; in other words, there are distracting behaviors such as making phone calls or talking with others.	Accident investigation and case analysis
HF4→UA12	Working in confined spaces with no air circulation for a long time, failing to breathe fresh air outside at intervals.	Accident investigation and case analysis
HF6→UA13	Failure to initially report dangerous situations to managers accurately and concretely.	Accident investigation and case analysis
**(B)**		
**Risk Factor**	**Description**	**Source**
HF18→UP1	There is insufficient awareness of the importance of safety or risk vigilance, and indifference to hidden hazards and emergencies.	[24,65]
HF20→UP2	The educational level, professional competence, and the ability of self-rescue and mutual rescue of operators are lacking.	[10,11]
HF22→UP3	Operators are unfamiliar with specific workplace and equipment operating procedures. They cannot think and act calmly in the face of emergencies.	[1,10,11]
HF17→UP4	The site conditions are complex, or the storage and transportation of toxic and harmful gases are improper, which can easily cause danger.	[1,24]
HF19→UP5	The ventilation conditions in the confined space are relatively poor and there are no safety placards or warning signs in the operation site.	[24,65]
HF8→UP6	The natural ventilation device fails to provide indoor air circulation and discharge of toxic and harmful gases, and the strong ventilation device is defective.	Accident investigation and case analysis
HF10→UP7	The safety valve of the device is not firm or activates accidentally; the connection of the pipeline is not tight.	Accident investigation and case analysis
HF12→UP8	The toxic and harmful gas pipelines are accidentally interconnected, leading to a sudden increase of local dangerous gases.	Accident investigation and case analysis
HF14→UP9	Safety devices such as sound and light alarms of various gas cylinders and pressure vessels are invalid, and personal protective equipment is lacking or defective.	Accident investigation and case analysis
**(C)**		
**Risk Factor**	**Description**	**Source**
HF26→US1	In the daily effort to address hidden dangers, there is a lack of lean-oriented management of key components of equipment and facilities and regular detection of the concentration of toxic and harmful gas, thus failure to find hidden dangers in time.	Expert experience
HF21→US2	Safety systems and procedures such as work certification, operation approval, and safety confirmation are not strictly implemented; and management personnel, especially on-site management personnel, fail to effectively implement supervision and management responsibilities.	[10,11,65]
HF23→US3	Lacking or improper coordination of key operation links, unreasonable operation personnel scheduling and teamwork, etc.	[1,11]
HF28→US4	Poor emergency response, thus failure to effectively organize escape and rescue operations.	Expert experience
HF30→US5	Improper storage of toxic and harmful gases, and complex and unsafe conditions of the workplace.	Expert experience
**(D)**		
**Risk Factor**	**Description**	**Source**
HF25→OI1	Insufficient briefing and training related to safety production, especially for new employees, and a lack of targeted first aid training on suffocation prevention.	Expert experience
HF27→OI2	There are language barriers in communicating with team members; and the awareness of teamwork and mutual assistance is poor.	Expert experience
HF29→OI3	The ‘people-oriented’ belief of safe production and the safety of organization members is not given enough attention.	Expert experience
HF31→OI4	The planning, operation, and supervision of ship repair systems are defective, especially in terms of procedures of daily safety management and emergency response.	Expert experience

**Table 3:** Criteria for calculating expert credibility.

Indicator	Classification	Score
Professional position	Senior academic/research fellow	5
	Junior academic/research fellow	4
	Engineer	3
	Technician	2
	Worker	1
Length of service (years)	≥30	5
	20–29	4
	10–19	3
	6–9	2
	≤5	1
Education level	Ph.D.	5
	Master’s degree	4
	B.S. or B.E.	3
	Junior college	2
	School level	1
Job title	Senior Captain Or Senior Chief Engineer	5
	Director/Captain Or Chief Engineer	4
	Department Manager or Chief Officer	3
	Manufacturing Supervisor	2
	Ratings	1

**Table 4:** The calculation results of expert reliability.

Expert	Professional Position	Length of Service	Education Level	Job Title	Weight
E1	Senior research fellow	≥30	Ph.D.	Director	0.2375
E2	Engineer	20–29	Master’s	Chief Officer	0.1750
E3	Junior academic	10–19	Master’s	Captain	0.1875
E4	Senior academic	20–29	Ph.D.	Senior Chief Engineer	0.2375
E5	Engineer	10–19	Master’s	Department Manager	0.1625

**Table 5:** Weight assignment of the identified human factors.

Indicator	*SV*	*IV*	*FP*	Indicator	*SV*	*IV*	*FP*
Errors	0.3324	— —	1.25 × 10^−^³	X9	0.1129	0.0390	2.01 × 10^−7^
Violations	0.3456	— —	1.42 × 10^−^³	X10	0.1085	0.0375	1.63 × 10^−7^
Environmental factors	0.3220	— —	1.12 × 10^−^³	X11	0.1163	0.0402	2.37 × 10^−7^
X1	0.2565	0.0853	8.41 × 10^−6^	X12	0.1122	0.0388	1.96 × 10^−7^
X2	0.2395	0.0796	6.27 × 10^−6^	X13	0.1000	0.0346	1.05 × 10^−7^
X3	0.2765	0.0919	1.15 × 10^−5^	X14	0.1619	0.0521	8.87 × 10^−7^
X4	0.2275	0.0756	5.01 × 10^−6^	X15	0.1665	0.0536	1.02 × 10^−6^
X5	0.1147	0.0396	2.19 × 10^−7^	X16	0.1648	0.0531	9.74 × 10^−7^
X6	0.1106	0.0382	1.80 × 10^−7^	X17	0.1748	0.0563	1.29 × 10^−6^
X7	0.1095	0.0378	1.70 × 10^−7^	X18	0.1724	0.0555	1.21 × 10^−6^
X8	0.1154	0.0399	2.28 × 10^−7^	X19	0.1596	0.0514	8.30 × 10^−7^

**Table 6:** Structural analysis under different states of the system.

**Failure**	**MCS Analysis**
MCS1–MCS33	{X1};{X10*X13*X14*X15*X16};{X10*X13*X14*X15*X17};{X10*X13*X14*X15*X18};{X10*X13*X14*X15*X19};{X11};{X12};{X2};{X3*X4};{X4*X10*X14*X15*X16};{X4*X10*X14*X15*X17};{X4*X10*X14*X15*X18};{X4*X10*X14*X15*X19};{X4*X6*X14*X15*X16};{X4*X6*X14*X15*X17};{X4*X6*X14*X15*X18};{X4*X6*X14*X15*X19};{X5};{X6*X13*X14*X15*X16};{X6*X13*X14*X15*X17};{X6*X13*X14*X15*X18};{X6*X13*X14*X15*X19};{X6*X7*X14*X15*X16};{X6*X7*X14*X15*X17};{X6*X7*X14*X15*X18};{X6*X7*X14*X15*X19};{X7*X10*X14*X15*X1};{X7*X10*X14*X15*X17};{X7*X10*X14*X15*X18};{X7*X10*X14*X15*X19};{X7*X13};{X8};{X9}
**Safe**	**MPS analysis**
MPS1–MPS17	{X1*X2*X3*X5*X6*X7*X8*X9*X10*X11*X12};{X1*X2*X3*X5*X6*X8*X9*X10*X11*X12*X13};{X1*X2*X3*X5*X7*X8*X9*X11*X12*X14};{X1*X2*X3*X5*X7*X8*X9*X11*X12*X15};{X1*X2*X3*X5*X7*X8*X9*X11*X12*X16*X17*X18*X19};{X1*X2*X3*X5*X8*X9*X11*X12*X13*X14};{X1*X2*X3*X5*X8*X9*X11*X12*X13*X15};{X1*X2*X3*X5*X8*X9*X11*X12*X13*X16*X17*X18*X19};{X1*X2*X4*X5*X6*X7*X8*X9*X10*X11*X12};{X1*X2*X4*X5*X6*X8*X9*X10*X11*X12*X13};{X1*X2*X4*X5*X7*X8*X9*X11*X12*X13};{X1*X2*X4*X5*X7*X8*X9*X11*X12*X14};{X1*X2*X4*X5*X7*X8*X9*X11*X12*X15};{X1*X2*X4*X5*X7*X8*X9*X11*X12*X16*X17*X18*X19};{X1*X2*X4*X5*X8*X9*X11*X12*X13*X14};{X1*X2*X4*X5*X8*X9*X11*X12*X13*X15};{X1*X2*X4*X5*X8*X9*X11*X12*X13*X16*X17*X18*X19}

## Data Availability

The data that support the findings of this study are available from the corresponding author upon reasonable request.

## Appendix A

**Table A1:** Pairwise comparison of the elements in level 2 of the direct causes that lead to the accident.

	Errors	Violations	Environmental Factors
Errors	(1,1,1) (1,1,1) (1,1,1) (1,1,1) (1,1,1)	(2/3,1,2) (1/2,2/3,1) (2/3,1,2) (1,1,1) (1,1,1)	(1,1,1) (1/2,1,3/2) (1/2,1,3/2) (1,1,1) (1,1,1)
Violations	(1/2,1,3/2) (1,3/2,2) (1/2,1,3/2) (1,1,1) (1,1,1)	(1,1,1) (1,1,1) (1,1,1) (1,1,1) (1,1,1)	(1/2,1,3/2) (1/2,1,3/2) (1,1,1) (1,1,1) (1,1,1)
Environmental factors	(1,1,1) (2/3,1,2) (2/3,1,2) (1,1,1) (1,1,1)	(2/3,1,2) (2/3,1,2) (1,1,1) (1,1,1) (1,1,1)	(1,1,1) (1,1,1) (1,1,1) (1,1,1) (1,1,1)

**Table A2:** Pairwise comparison of the elements in the category of ‘Errors’.

	X1	X2	X3	X4
X1	(1,1,1) (1,1,1) (1,1,1) (1,1,1) (1,1,1)	(1 3/2 2) (1,1,1) (1,1,1) (1/2 1 3/2) (1 3/2 2)	(2/3,1,2) (2/3,1,2) (1/2,2/3,1) (1/2,2/3,1) (1/2,1,3/2)	(1/2,1,3/2) (1/2,1,3/2) (2/3,1,2) (1,1,1) (1/2,2/3,1)
X2	(1/2,2/3,1) (1,1,1) (1,1,1) (2/3,1,2) (1/2,2/3,1)	(1,1,1) (1,1,1) (1,1,1) (1,1,1) (1,1,1)	(2/3,1,2) (2/3,1,2) (2/3,1,2) (1/2,1,3/2) (1/2,1,3/2)	(1,1,1) (1/2,1,3/2) (2/3,1,2) (2/3,1,2) (1/2,1,3/2)
X3	(1/2,1,3/2) (1/2,1,3/2) (1,3/2,2) (1,3/2,2) (2/3,1,2)	(1/2,1,3/2) (1/2,1,3/2) (1/2,1,3/2) (2/3,1,2) (2/3,1,2)	(1,1,1) (1,1,1) (1,1,1) (1,1,1) (1,1,1)	(1,3/2,2) (3/2,2,5/2) (1,3/2,2) (3/2,2,5/2) (1,1,1)
X4	(2/3,1,2) (2/3,1,2) (1/2,1,3/2) (1,1,1) (1,3/2,2)	(1,1,1) (2/3,1,2) (1/2,1,3/2) (1/2,1,3/2) (2/3,1,2)	(1/2,2/3,1) (2/5,1/2,2/3) (1/2,2/3,1) (2/5,1/2,2/3) (1,1,1)	(1,1,1) (1,1,1) (1,1,1) (1,1,1) (1,1,1)

**Table A3:** Pairwise comparison of the elements in the category of ‘Violations’.

	X5	X6	X7	X8	X9	X10	X11	X12	X13
X5	(1,1,1) (1,1,1) (1,1,1) (1,1,1) (1,1,1)	(1,1,1) (2/3,1,2) (1/2,2/3,1) (1/2,1,3/2) (1,3/2,2)	(1,3/2,2) (1/2,2/3,1) (2/3,1,2) (2/3,1,2) (1/2,1,3/2)	(2/3,1,2) (2/3,1,2) (1,1,1) (1,1,1) (3/2,2,5/2)	(2/5,1/2,2/3) (2/3,1,2) (2/3,1,2) (2/3,1,2) (2/3,1,2)	(1/2,1,3/2) (1/2,1,3/2) (1/2,1,3/2) (1,1,1) (1,1,1)	(1/2,2/3,1) (1/2,2/3,1) (2/3,1,2) (2/3,1,2) (2/5,1/2,2/3)	(1/2,1,3/2) (1/2,2/3,1) (1/2,2/3,1) (1,3/2,2) (1,3/2,2)	(3/2,2,5/2) (1,3/2,2) (3/2,2,5/2) (1,3/2,2) (3/2,2,5/2)
X6	(1,1,1) (1/2,1,3/2) (1,3/2,2) (2/3,1,2) (1/2,2/3,1)	(1,1,1) (1,1,1) (1,1,1) (1,1,1) (1,1,1)	(1/2,1,3/2) (1/2,1,3/2) (3/2,2,5/2) (1/2,1,3/2) (1/2,1,3/2)	(1/2,1,3/2) (1/2,1,3/2) (1,3/2,2) (1/2,1,3/2) (1/2,1,3/2)	(1/2,1,3/2) (1,1,1) (1,1,1) (1/2,2/3,1) (2/3,1,2)	(1/2,1,3/2) (1/2,1,3/2) (1/2,1,3/2) (1/2,1,3/2) (1/2,1,3/2)	(1/2,2/3,1) (1/3,2/5,1/2) (2/3,1,2) (2/3,1,2) (2/3,1,2)	(1,1,1) (1/2,1,3/2) (1/2,1,3/2) (1/2,1,3/2) (2/5,1/2,2/3)	(1/2,1,3/2) (1/2,1,3/2) (3/2,2,5/2) (1/2,1,3/2) (1,3/2,2)
X7	(1/2,2/3,1) (1,3/2,2) (1/2,1,3/2) (1/2,1,3/2) (2/3,1,2)	(2/3,1,2) (2/3,1,2) (2/5,1/2,2/3) (2/3,1,2) (2/3,1,2)	(1,1,1) (1,1,1) (1,1,1) (1,1,1) (1,1,1)	(2/3,1,2) (2/3,1,2) (1/2,1,3/2) (2/3,1,2) (1,3/2,2)	(1/2,1,3/2) (1/2,1,3/2) (1/2,2/3,1) (1/2,1,3/2) (2/3,1,2)	(2/3,1,2) (2/3,1,2) (2/3,1,2) (2/3,1,2) (1,1,1)	(1/2,2/3,1) (2/3,1,2) (2/3,1,2) (1,1,1) (1/2,1,3/2)	(2/3,1,2) (2/3,1,2) (1/2,1,3/2) (2/3,1,2) (2/3,1,2)	(1,3/2,2) (1/2,1,3/2) (1/2,1,3/2) (1/2,1,3/2) (1,1,1)
X8	(1/2,1,3/2) (1/2,1,3/2) (1,1,1) (1,1,1) (2/5,1/2,2/3)	(2/3,1,2) (2/3,1,2) (1/2,2/3,1) (2/3,1,2) (2/3,1,2)	(1/2,1,3/2) (1/2,1,3/2) (2/3,1,2) (1/2,1,3/2) (1/2,2/3,1)	(1,1,1) (1,1,1) (1,1,1) (1,1,1) (1,1,1)	(1,3/2,2) (1/2,1,3/2) (1,3/2,2) (1/2,1,3/2) (1/2,1,3/2)	(2/3,1,2) (1,3/2,2) (1,3/2,2) (2/3,1,2) (3/2,2,5/2)	(2/3,1,2) (2/3,1,2) (2/3,1,2) (1/2,1,3/2) (1/2,1,3/2)	(1/2,1,3/2) (2/3,1,2) (1/2,1,3/2) (1/2,1,3/2) (1/2,1,3/2)	(1,3/2,2) (1,3/2,2) (1,3/2,2) (1,3/2,2) (1,3/2,2)
X9	(3/2,2,5/2) (1/2,1,3/2) (1/2,1,3/2) (1/2,1,3/2) (1/2,1,3/2)	(2/3,1,2) (1,1,1) (1,1,1) (1,3/2,2) (1/2,1,3/2)	(2/3,1,2) (2/3,1,2) (1,3/2,2) (2/3,1,2) (1/2,1,3/2)	(1/2,2/3,1) (2/3,1,2) (1/2,2/3,1) (2/3,1,2) (2/3,1,2)	(1,1,1) (1,1,1) (1,1,1) (1,1,1) (1,1,1)	(1/2,1,3/2) (1,3/2,2) (1,3/2,2) (1/2,1,3/2) (1/2,1,3/2)	(2/3,1,2) (2/3,1,2) (2/3,1,2) (1/2,1,3/2) (1/2,1,3/2)	(1/2,1,3/2) (1/2,2/3,1) (1/2,1,3/2) (1/2,1,3/2) (1,3/2,2)	(1/2,1,3/2) (1/2,1,3/2) (1,1,1) (1,1,1) (2/3,1,2)
X10	(2/3,1,2) (2/3,1,2) (2/3,1,2) (1,1,1) (1,1,1)	(2/3,1,2) (2/3,1,2) (2/3,1,2) (2/3,1,2) (2/3,1,2)	(1/2,1,3/2) (1/2,1,3/2) (1/2,1,3/2) (1/2,1,3/2) (1,1,1)	(1/2,1,3/2) (1/2,2/3,1) (1/2,2/3,1) (1/2,1,3/2) (2/5,1/2,2/3)	(2/3,1,2) (1/2,2/3,1) (1/2,2/3,1) (2/3,1,2) (2/3,1,2)	(1,1,1) (1,1,1) (1,1,1) (1,1,1) (1,1,1)	(1/2,2/3,1) (2/3,1,2) (1/2,1,3/2) (1,1,1) (1,1,1)	(1/2,1,3/2) (1/2,1,3/2) (1/2,1,3/2) (2/3,1,2) (2/3,1,2)	(1/2,1,3/2) (1/2,1,3/2) (3/2,2,5/2) (1,3/2,2) (1/2,1,3/2)
X11	(1,3/2,2) (1,3/2,2) (1/2,1,3/2) (1/2,1,3/2) (3/2,2,5/2)	(1,3/2,2) (2,5/2,3) (1/2,1,3/2) (1/2,1,3/2) (1/2,1,3/2)	(1,3/2,2) (1/2,1,3/2) (1/2,1,3/2) (1,1,1) (2/3,1,2)	(1/2,1,3/2) (1/2,1,3/2) (1/2,1,3/2) (2/3,1,2) (2/3,1,2)	(1/2,1,3/2) (1/2,1,3/2) (1/2,1,3/2) (2/3,1,2) (2/3,1,2)	(1,3/2,2) (1/2,1,3/2) (1/2,1,3/2) (1/2,1,3/2) (1/2,1,3/2)	(1,1,1) (1,1,1) (1,1,1) (1,1,1) (1,1,1)	(1/2,1,3/2) (2/3,1,2) (2/3,1,2) (1/2,1,3/2) (1,1,1)	(1/2,1,3/2) (1/2,1,3/2) (1,1,1) (1/2,1,3/2) (1/2,1,3/2)
X12	(2/3,1,2) (1,3/2,2) (1,3/2,2) (1/2,2/3,1) (1/2,2/3,1)	(1,1,1) (2/3,1,2) (2/3,1,2) (2/3,1,2) (3/2,2,5/2)	(1/2,1,3/2) (1/2,1,3/2) (2/3,1,2) (1/2,1,3/2) (1/2,1,3/2)	(2/3,1,2) (1/2,1,3/2) (2/3,1,2) (2/3,1,2) (2/3,1,2)	(2/3,1,2) (1,3/2,2) (2/3,1,2) (2/3,1,2) (1/2,2/3,1)	(2/3,1,2) (2/3,1,2) (2/3,1,2) (1/2,1,3/2) (1/2,1,3/2)	(2/3,1,2) (1/2,1,3/2) (1/2,1,3/2) (2/3,1,2) (1,1,1)	(1,1,1) (1,1,1) (1,1,1) (1,1,1) (1,1,1)	(1,3/2,2) (1/2,1,3/2) (1/2,1,3/2) (1/2,1,3/2) (1/2,1,3/2)
X13	(2/5,1/2,2/3) (1/2,2/3,1) (2/5,1/2,2/3) (1/2,2/3,1) (2/5,1/2,2/3)	(2/3,1,2) (2/3,1,2) (2/5,1/2,2/3) (2/3,1,2) (1/2,2/3,1)	(1/2,2/3,1) (2/3,1,2) (2/3,1,2) (2/3,1,2) (1,1,1)	(1/2,2/3,1) (1/2,2/3,1) (1/2,2/3,1) (1/2,2/3,1) (1/2,2/3,1)	(2/3,1,2) (2/3,1,2) (1,1,1) (1,1,1) (1/2,1,3/2)	(2/3,1,2) (2/3,1,2) (2/5,1/2,2/3) (1/2,2/3,1) (2/3,1,2)	(2/3,1,2) (2/3,1,2) (1,1,1) (2/3,1,2) (2/3,1,2)	(1/2,2/3,1) (2/3,1,2) (2/3,1,2) (2/3,1,2) (2/3,1,2)	(1,1,1) (1,1,1) (1,1,1) (1,1,1) (1,1,1)

**Table A4:** Pairwise comparison of the elements in the category of ‘Environmental factors’.

	X14	X15	X16	X17	X18	X19
X14	(1,1,1) (1,1,1) (1,1,1) (1,1,1) (1,1,1)	(2/3,1,2) (1/2,1,3/2) (1/2,1,3/2) (2/3,1,2) (2/3,1,2)	(2/3,1,2) (2/3,1,2) (2/3,1,2) (1,1,1) (2/3,1,2)	(1/2,2/3,1) (2/3,1,2) (2/3,1,2) (2/3,1,2) (2/5,1/2,2/3)	(2/3,1,2) (2/3,1,2) (2/3,1,2) (1/2,2/3,1) (2/3,1,2)	(1/2,1,3/2) (1,1,1) (1/2,1,3/2) (1/2,1,3/2) (2/3,1,2)
X15	(1/2,1,3/2) (2/3,1,2) (2/3,1,2) (1/2,1,3/2) (1/2,1,3/2)	(1,1,1) (1,1,1) (1,1,1) (1,1,1) (1,1,1)	(2/3,1,2) (1/2,1,3/2) (1/2,1,3/2) (2/3,1,2) (2/3,1,2)	(2/3,1,2) (1/2,2/3,1) (1/2,2/3,1) (2/3,1,2) (2/3,1,2)	(2/3,1,2) (1,1,1) (2/3,1,2) (2/3,1,2) (2/3,1,2)	(1/2,1,3/2) (1,3/2,2) (1,3/2,2) (1/2,1,3/2) (2/3,1,2)
X16	(1/2,1,3/2) (1/2,1,3/2) (1/2,1,3/2) (1,1,1) (1/2,1,3/2)	(1/2,1,3/2) (2/3,1,2) (2/3,1,2) (1/2,1,3/2) (1/2,1,3/2)	(1,1,1) (1,1,1) (1,1,1) (1,1,1) (1,1,1)	(2/3,1,2) (2/3,1,2) (1/2,2/3,1) (2/3,1,2) (1,1,1)	(2/3,1,2) (1/2,2/3,1) (2/3,1,2) (2/3,1,2) (1/2,1,3/2)	(1,3/2,2) (1/2,1,3/2) (1/2,1,3/2) (1/2,1,3/2) (1/2,1,3/2)
X17	(1,3/2,2) (1/2,1,3/2) (1/2,1,3/2) (1/2,1,3/2) (3/2,2,5/2)	(1/2,1,3/2) (1,3/2,2) (1,3/2,2) (1/2,1,3/2) (1/2,1,3/2)	(1/2,1,3/2) (1/2,1,3/2) (1,3/2,2) (1/2,1,3/2) (1,1,1)	(1,1,1) (1,1,1) (1,1,1) (1,1,1) (1,1,1)	(1,3/2,2) (2/3,1,2) (2/3,1,2) (1/2,1,3/2) (1/2,1,3/2)	(1/2,1,3/2) (1/2,1,3/2) (1/2,1,3/2) (1/2,1,3/2) (1,3/2,2)
X18	(1/2,1,3/2) (1/2,1,3/2) (1/2,1,3/2) (1,3/2,2) (1/2,1,3/2)	(1/2,1,3/2) (1,1,1) (1/2,1,3/2) (1/2,1,3/2) (1/2,1,3/2)	(1/2,1,3/2) (1,3/2,2) (1/2,1,3/2) (1/2,1,3/2) (2/3,1,2)	(2/3,1,2) (1/2,1,3/2) (1/2,1,3/2) (2/3,1,2) (2/3,1,2)	(1,1,1) (1,1,1) (1,1,1) (1,1,1) (1,1,1)	(1,3/2,2) (1/2,1,3/2) (1/2,1,3/2) (1/2,1,3/2) (3/2,2,5/2)
X19	(2/3,1,2) (1,1,1) (2/3,1,2) (2/3,1,2) (1/2,1,3/2)	(2/3,1,2) (1/2,2/3,1) (1/2,2/3,1) (2/3,1,2) (1/2,1,3/2)	(1/2,2/3,1) (2/3,1,2) (2/3,1,2) (2/3,1,2) (2/3,1,2)	(2/3,1,2) (2/3,1,2) (2/3,1,2) (2/3,1,2) (1/2,2/3,1)	(1/2,2/3,1) (2/3,1,2) (2/3,1,2) (2/3,1,2) (2/5,1/2,2/3)	(1,1,1) (1,1,1) (1,1,1) (1,1,1) (1,1,1)
